# Automatic ladybird beetle detection using deep-learning models

**DOI:** 10.1371/journal.pone.0253027

**Published:** 2021-06-10

**Authors:** Pablo Venegas, Francisco Calderon, Daniel Riofrío, Diego Benítez, Giovani Ramón, Diego Cisneros-Heredia, Miguel Coimbra, José Luis Rojo-Álvarez, Noel Pérez

**Affiliations:** 1 Colegio de Ciencias e Ingenierías “El Politécnico”, Universidad San Francisco de Quito USFQ, Quito, Ecuador; 2 Museo de Zoología, Instituto iBIOTROP & Colegio de Ciencias Biológicas y Ambientales COCIBA, Universidad San Francisco de Quito USFQ, Quito, Ecuador; 3 INESC TEC, Faculdade de Ciências da Universidade do Porto, Porto, Portugal; 4 Department of Signal Theory and Communications and Telematic Systems and Computation, Rey Juan Carlos University, Fuenlabrada, Spain; Vellore Institute of Technology: VIT University, INDIA

## Abstract

Fast and accurate taxonomic identification of invasive trans-located ladybird beetle species is essential to prevent significant impacts on biological communities, ecosystem functions, and agricultural business economics. Therefore, in this work we propose a two-step automatic detector for ladybird beetles in random environment images as the first stage towards an automated classification system. First, an image processing module composed of a saliency map representation, simple linear iterative clustering superpixels segmentation, and active contour methods allowed us to generate bounding boxes with possible ladybird beetles locations within an image. Subsequently, a deep convolutional neural network-based classifier selects only the bounding boxes with ladybird beetles as the final output. This method was validated on a 2, 300 ladybird beetle image data set from Ecuador and Colombia obtained from the *iNaturalist* project. The proposed approach achieved an accuracy score of 92% and an area under the receiver operating characteristic curve of 0.977 for the bounding box generation and classification tasks. These successful results enable the proposed detector as a valuable tool for helping specialists in the ladybird beetle detection problem.

## Introduction

Insects are the most diverse group of animals, with more than 1 million described species [1]. They are key components of Earth ecosystems and provide invaluable services for humanity [2]. Recent studies have shown steep declines in insect diversity and population worldwide. Thus, taxonomic information increment has become more urgent than ever to develop insect-efficient conservation efforts [3]. However, efficient taxonomic identification of insects is challenging due to the high richness of some taxonomic groups, significant inter and intraspecific morphological variation, diverse life histories, complex distributions, and the decline of trained experts able to provide reliable identifications [4].

Ladybird beetles, which are members of the *Coccinellidae* family, are among the most distinctive and widespread groups of insects due to their colorful patterns and reputation as agricultural pest controllers [5]. There are more than 6, 000 described species of ladybird beetles. However, its diversity is still far from understood in the Neotropics, where new genera and species are frequently described, especially in countries such as Ecuador where large areas remain unexplored due to few specialists working on the field [6]. Although the *Coccinellidae* family includes some species feeding on plants and fungi, many ladybird beetles are top predators in terrestrial invertebrate communities [5]. Several species of ladybird beetles are important predators of agricultural pests, such as aphids, scale insects, and whiteflies. They have been deliberately translocated as biological control agents across the world since the late 19^th^ century [5].

Translocated ladybird beetles, such as *Harmonia axyridis* and *Coccinella septempunctata*, have established naturalized and expanding populations, becoming invasive and having a significant impact on biological communities, ecosystem functions, and agribusinesses economies [5]. *Harmonia axyridis*, native to East Asia, nowadays has established populations in America, Europe, Africa, and New Zealand, and it is considered the most invasive ladybird on Earth [7]. Despite predictions of its potential invasive expansion, the presence of *Harmonia axyridis* and other non-native ladybird beetles in many countries of the Global South has usually been reported after populations are well-established [8]. Statistically, the use of citizen science records has proved exceptionally useful to discover new populations [9]. Morphologically, species identification is particularly applicable in organisms with distinctive coloration patterns. Although several members of the *Coccinellidae* family lack distinctive patterns, many predaceous ladybird beetles, including those commonly used as biological control agents, are brightly colored [10].

New technologies, such as machine learning techniques and public participation in research through citizen science, provide crucial opportunities for developing tools that can aid to increase the discovery and identification of insect diversity significantly [11, 12]. These tools might also offer advances for early identification and detection of non-native translocated species, thus allowing for the establishment of monitoring, management, and control programs of invasive species [13].

Machine learning classifiers (MLC) based on shallow and deep learning have been widely used for object detection and classification in different scenarios [14]. For example, in medical applications [15–18], volcanology [19–22], surveillance and security [23], intelligent transportation systems [24], energy and materials saving [25], or marine ecosystems [26, 27], among many others. In the context of insect detection and classification, several machine learning approaches have been developed [28–32]. Some of them include the feature calculation, the feature selection, or the space reduction tasks before the classification step in order to moderate the classifier complexity. Others exhaustively explore the whole image attempting the detection and classification tasks but this increases the model complexity.

Despite these advances, research regarding the automatic detection and classification of ladybird beetles is scarce in the literature. Examples using deep learning classifiers as in [28, 31, 33] provide good performance but incur in high computational costs as they skip the classification space reduction, for example, sometimes preferring to slide a subwindow into the whole image to attempt any detection or classification task. On the other hand, classifiers based on shallow learning as in [34, 35] are less complex but with inferior performances. Manual identification by experts is still used as the reliable approach. However, this mechanism is always prone to errors introduction due to fatigue and workload, so that the problem of automatic ladybird beetle detection and classification remains challenging.

Therefore, this work proposes a new automatic detector based on the combination of digital image processing and deep-learning techniques to maximize the detection performance of ladybird beetles on random environment images. First, possible regions with ladybird beetles inside the image are detected. Subsequently, these regions are classified by a deep convolutional neural network (CNN) model, which determines which of them contains ladybird beetles or not. The principal advantage and novelty of this approach is reducing the classification space to decrease the complexity required from the classification model while maximizing its performance. Accordingly, the main contributions in this proposal are related to:

- *Detection of suspected regions*: We combine three digital image processing methods such as saliency map, simple linear iterative clustering (SLIC) superpixels segmentation, and active contour to determine possible regions with ladybird beetles inside. These methods operate over the whole image but reduce the space at each stage to generate the suspected bounding boxes.
- *Reduced classification space*: We obtain a set of bounding boxes (suspected regions) from each image under analysis, where each of them is smaller than the input image. Therefore, our classification space is substantially reduced versus the original one.
- *Deep learning classification strategy*: We take advantage of the reduced classification space to explore and optimize several deep CNN models. This strategy allowed us to successfully classify the suspected set of bounding boxes with the most suitable classification model.

Furthermore, this work constitutes an initial step towards an automated classification system that can help the specialists detect endemic and invasive species quickly and accurately.

The rest of the paper is organized as follows. The Related Work Section briefly describes previously developed approaches in the context of insect classification. The Materials and Methods Section presents the employed database, together with a brief description of employed digital image processing methods such as Saliency map, SLIC superpixels segmentation, active contour, and deep CNN architectures. Also, a detailed description of the proposed detector and the experimental setup designed for the detector evaluation is included. The Results and Discussion Section outlines the accuracy (ACC) results in the bounding box generation. The classification output is validated based on the area under the receiver operating characteristic curve (AUC) scores obtained by the selected deep CNN models using the Wilcoxon statistical test [36] for evaluating the importance of the differences between the classification models. The limitations of the proposed detector are presented, and finally, Conclusions and Future Work are summarized in the last section.

### Related work

During the last decade, several approaches based on shallow and deep learning have been developed to tackle the problem of insect detection and classification in random environments. For example, in [28], a mobile application was built to classify 30 kinds of forest insects using a CNN. The network was validated on a 29, 722 sample data set, obtaining an ACC score of 94%. In [37], a morphometric analysis of beetles was conducted by detecting landmarks on beetle images with a CNN classifier. The method was validated on a data set of 293 samples, reaching an ACC score of 78.79%. Similarly, in [29], a morphological analysis of biting midges wing was carried out to discern among four different species. The linear discriminant analysis model was the best classifier on a data set with 192 samples, obtaining an AUC score of 0.96.

In [30], a speeded-up robust features (SURF) extraction method was combined with a support vector machine (SVM) classifier to recognize 102 species of insect pest. The method reached a low ACC score of 19.5% on a data set with 75, 000 samples. In [38], a combination of sparse-coding histogram features and multiple kernel learning techniques were employed to classify 24 insect species of field crops such as corn, soybeans, wheat, and canola. The model obtained an ACC score of 85.5% on a data set of 600 samples. A similar ACC value of 85% was reached by a sequential minimal optimization SVM classifier while processing 35 different moth species from UK territory on a data set with 774 samples [39]. In [40], an SVM classifier with radial basis kernel function was used to identify four species of rice pests on a data set with 156 feature vectors, reaching an ACC score of 97.5%. In [34], a set of color and geometrical features was computed to classify 360 images of ladybird beetles using a probabilistic neural network-based classifier, achieving a mean ACC value of 88.19%. Likewise, the developed method in [35] employed a combination of a multilayer perceptron and a J48 decision tree to classify 9 species of ladybird beetles, obtaining an averaged ACC of 81.93%.

Furthermore, some deep learning approaches have been used for recognizing varieties of insect pests, such as in [41], where the InceptionV3 model was modified to recognize six different pests of maize plantations, reaching an ACC score of 49, 7%. In [30], a combination of a deep CNN ResNet and an SVM model was used for feature extraction and classification of insect pest species, respectively, obtaining an ACC value of 49.5%. Similarly, in [31], dense scale-invariant features and a deep CNN model was employed to classify brown plant-hoppers and ladybirds in rice crops, attaining an ACC score of 97%. In [42], a combination of You Only Look Once (YOLO) and SVM models were employed to segment and classify six species of flying insects, reaching ACC scores of 93.71% and 92.50% in the segmentation and classification stages, respectively. Similarly, in [32], a modified U-net model with a simplified VGG16 network was proposed to segment butterflies from ecological images, obtaining an ACC score of 98.67%. Lately, 18 different ladybird beetle species from the UK were classified using a CNN-ResNet model, achieving an overall ACC of 69% [33].

Given the background summarized in this section, it is possible to notice that most previously developed methods were employed to tackle the insect detection and classification problem in a general way. Only a few of them were applied to detect and classify ladybird beetles, but obtaining reduced performance as in [34, 35, 37] or incurring in a high model cost of classification as in [33]. Therefore, we expect that the contributions of the proposed method address the existing limitations of developing automatic ladybird beetle detection models without a high classification cost and without losing detection performance.

## Materials and methods

### Database

This work used a ladybird beetle image database taken from the publicly available *iNaturalist* project, which is provided by courtesy of the California Academy of Sciences and the National Geographic Society, and it is available at http://www.inaturalist.org. This project consists of an online initiative where observations of different animal species are well documented by the general public and specialists worldwide. Also, using the implemented search engine makes it possible to filter the data according to their taxonomic categories and select the desired species samples.

We only assembled ladybird beetle data from Ecuador and Colombia regions labeled as *research-grade*, which means that the observations were verified by experts in the *iNaturalist* project. Moreover, we individually inspected each observation to confirm correct family identification and keep only adult samples. Adult ladybird beetles have external diagnostic morphology and coloration patterns, allowing their identification in random environment images. Therefore, the employed dataset contains a total of 2, 300 images with different sizes but including at least one identifiable ladybird beetle sample within the image. It is important to note that, as the photos correspond to general observations reported by users to the *iNaturalist* project without any format restriction, the location and the ladybird beetles size within the image, as well as image sizes, vary significantly across the dataset.

### Saliency map

A saliency map is an image representation that shows where attention focus is given in a scene by the unique quality of each pixel. Thus, its output is a pixel subset of the image, which is subsequently easier to analyze. This type of image segmentation reveals the most relevant areas in a picture using as a basis the spatial organization of features of the image [43]. Finding salient regions of an image helps various tasks in computer vision, such as speeding up object detection [44], object recognition [45], object tracking [46], and content-aware image editing [47].

Among several established approaches [48, 49], Kanan and Cottrell [50] implemented an algorithm which is biologically inspired to model visual attention by relying on two facets of the optical system. The sparse visual features that capture the statistical regularities in natural scenes such as luminance, color, contrast, blur, edges, and the sequential fixation-based visual attention attempt to mimic the way we sequentially look at salient locations of an object in a scene.

The sparse visual features are computed by applying independent component analysis (ICA) filters to image patches to produce a set of sparse filters with luminance and chromatic properties similar to simple cells in the primate visual cortex [51]. The saliency map model (*P*(*f*)), using ICA features, is defined using a generalized Gaussian distribution (GGD) given by:

$$\begin{array}{c} P\left(f_{i}\right)=\frac{\theta_{i}}{2\sigma_{i}\Gamma \left(\theta_{i}^{-1}\right)}exp(-{\left|\frac{f_{i}}{\sigma_{i}}\right|}^{\theta_{i}}) \end{array}$$ (1)

where *f* indicates the ICA features, *f*_*i*_ is the *i*^*th*^ element of the vector *f*; *θ*_*i*_ and *σ*_*i*_ are the shape and scale parameters of the GDD respectively, and Γ is the gamma function. These parameters were estimated with the algorithm proposed by Song [52], which assumes that *θ* and Γ are independent. Thus, additional subroutines are not needed to evaluate them, making this algorithm a practical optimizer of *P*(*f*).

On the other hand, during the fixation stage, the preliminary saliency map (obtained in the previous step) is normalized to be a probability distribution constrained to one (unitary constrain). This distribution is used to compute the fixations, which are windows with variable sizes located over the most important regions in the image under analysis. Subsequently, according to Eq (1), the process of saliency map computation is applied iteratively until exploring all fixation windows. Finally, the preliminary saliency map and all saliency maps computed from the fixation windows are merged to form the final output (saliency map image).

### SLIC superpixels segmentation

Superpixels algorithms cluster pixels that share similar qualities into multiple sets of pixels (superpixel segments). The goal is to simplify the segmentation process by changing the representation of an image into another which is easier to analyze. These algorithms have become key building blocks of many computer vision systems to reduce the complexity of subsequent image processing tasks. There are many approaches to superpixels segmentation algorithms [53–56]. The simple linear iterative clustering (SLIC) [57] stands as a practical option to be considered in this work since it has been demonstrated to be faster and more memory efficient than existing methods. Additionally, it offers flexibility in the compactness and number of superpixels that it generates [58].

The SLIC superpixel algorithm is an adaptation of the k-means method for superpixel generation. It is based on the color and spatial proximity of the pixels in the image plane. The algorithm transforms the images to the CIELAB color space, in which each color is composed of three components: L*, a*, b*, representing the lightness (L*), the color tone from green to red (a*), and the color tone from blue to yellow (b*), respectively. Additionally, the pixel position information is represented as an [*x*, *y*] vector of coordinates. The SLIC superpixel algorithm merges both the value of the components [L*, a*, b*] and the pixel position into a five-dimensional vector [L*, a*, b*, *x*, *y*] representing the information of the current pixel under analysis in the feature space. The algorithm then computes distances (*D*) among pixels in the whole space to create superpixel segments and determine their size and compactness. However, the application of *D* can not be defined just by a five-dimensional Euclidean distance because, for large superpixel segments, the spatial distance outweighs the color proximity. Therefore, the color and spatial proximity must be normalized with respect to their maximum distances. The improved distance equation of *D* is given by:

$$\begin{array}{c} D=\sqrt{{\left(\frac{d_{c}}{M_{c}}\right)}^{2}+{\left(\frac{d_{s}}{M_{s}}\right)}^{2}} \end{array}$$ (2)

subject to:

$$\begin{array}{cc} d_{c}= & \sqrt{{\left(L_{j}-L_{i}\right)}^{2}+{\left(a_{j}-a_{i}\right)}^{2}+{\left(b_{j}-b_{i}\right)}^{2}}\\ d_{s}= & \sqrt{{\left(x_{j}-x_{i}\right)}^{2}+{\left(y_{j}-y_{i}\right)}^{2}} \end{array}$$

where *d*_*c*_ and *d*_*s*_ are the color and spatial distances between the *i* and *j* pixels, and *M*_*c*_ and *M*_*s*_ their maximum distance scores, respectively. It must be considered that *M*_*s*_ within the clusters (superpixel segments) should correspond to the sampling interval $M_{s}=\sqrt{\left(\frac{n}{k}\right)}$, being *n* and *k* the number of pixels and superpixels, respectively.

### Active contour

Active contour models, also known as snakes or deformable models, are algorithms based on an energy-minimizing curve that consider external constraints and image forces to determine lines and edges [59]. These models are generally used in computer vision to refine the delineation of an object systematically. Although commonly used in image segmentation, these algorithms can not identify objects in an image by themselves since they require an initial contour that serves as a seed to initialize the outline refining process (curve deformation).

In this work, we used the Chan-Vese’s active contour algorithm [60]. In contrast to the classical models that rely on the stopping-edge function based on the image gradient, the Chan-Vese algorithm treats the segmentation as an energy minimization problem with the stopping-edge function based on Mumford–Shah segmentation techniques [61]. This modification enables this model to discover contours both with or without gradient, thus providing advantages in detecting object contours in very noisy images, in cases where objects have very smooth boundaries, or even with discontinuous boundaries.

An overview of the mathematical formulation of the Chan-Vese model starts with the original snake model of Kass and Terzopoulos [59], which is subject to constraints of an input image (*u*_0_), e.g., the user initializes a curve around the desired object in the image. It moves until reaching the object boundary, and their formula is given by:

$$\begin{array}{c} J\left(C\right)=\alpha \int_{0}^{1}{\left|C^{\prime }\left(s\right)\right|}^{2}ds+\beta \int_{0}^{1}|C^{\prime \prime }\left(s\right)|ds-\lambda \int_{0}^{1}{\left|\nabla u_{0}\left(C\left(s\right)\right)\right|}^{2}ds \end{array}$$ (3)

where *C* is a parameterized curve on the gradient of the input image *u*_0_ and *α*, *β*, and λ are positive coefficients. The first two terms of the equation carry out the curve smoothness (internal energy), while the third term represents the curve attraction toward the objects in the image (external energy). It should be noted that by minimizing the energy (in Eq (3)), the curve acts as an edge detector by positioning at the points of maxima ∇*u*_0_(*C*(*s*)) while keeping a smoothness in the curve (object boundary). In contrast, the curve energy minimization of Mumford–Shah is a functional technique that establishes an optimal criterion for segmenting the image objects. It consists of minimizing:

$$\begin{array}{c} \begin{array}{cc} F^{MS}\left(u,C\right) & =\mu \cdot \xi \left(C\right)+\lambda \int_{\Omega }^{}{\left|u_{0}\left(x,y\right)-u\left(x,y\right)\right|}^{2}dxdy\\ & \;+\int_{\Omega \backslash C}^{}{\left|\nabla u(x,y)\right|}^{2}dxdy \end{array} \end{array}$$ (4)

subject to:

$$\begin{array}{c} \xi \left(C\right)=\int_{\Omega }^{}\delta_{0}\left(\phi \left(x,y\right)\right)|\nabla \phi (x,y)|dxdy \end{array}$$

where *μ* and λ (as in Eq 3) are positive coefficients, *u* is the best approximation of *u*_0_ (average inside or outside of *C*), *Ω* is the domain of application, *δ*_0_ is the one-dimensional Dirac measure, *ϕ* is the evolving curve *C*, and *ξ* is the length of curve *C*.

**Fig 1:**
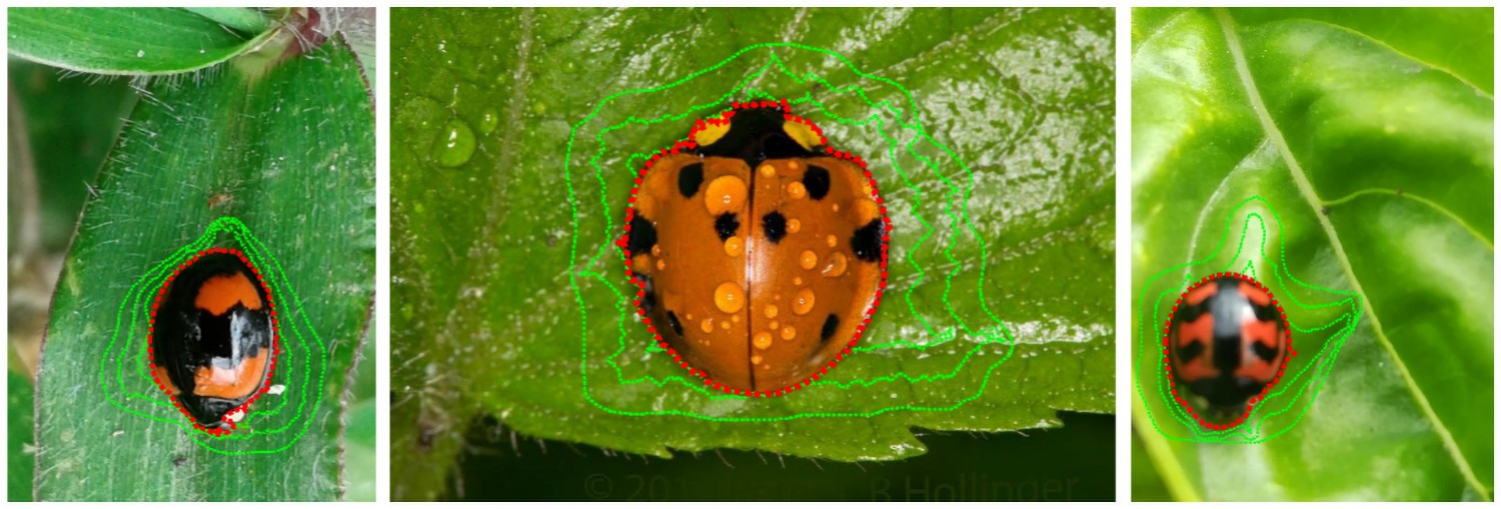
Deformation example of the Chan-Vese active contour curve (green lines) until reaching the ladybird beetles boundary (red line) on the image.

Finally, from the Kass and Terzopoulos original snake model (Eq (3)) and Mumford–Shah functional (Eq (4)), Chan-Vese proposed an active contour model, which does not depend on the gradient of the image to find the object boundary. This model is defined as:

$$\begin{array}{c} \begin{array}{cc} F(c_{1},c_{2},\phi ) & =\mu \int_{\Omega }^{}\delta \left(\phi \left(x,y\right)\right)|\nabla \phi (x,y)|dxdy+\nu \int_{\Omega }^{}H\left(\phi \left(x,y\right)\right)dxdy\\ & \;+\lambda_{1}\int_{\Omega }^{}{\left|u_{0}\left(x,y\right)-c_{1}\right|}^{2}H\left(\phi \left(x,y\right)\right)dxdy\\ & \;+\lambda_{2}\int_{\Omega }^{}{\left|u_{0}\left(x,y\right)-c_{2}\right|}^{2}(1-H\left(\phi \left(x,y\right)\right)dxdy \end{array} \end{array}$$ (5)

where *C*_1_ and *C*_2_ are constants given by the average of *u*_0_ inside and outside the evolving curve *ϕ*, respectively. *H* is the Heaviside function and *μ* (as in Eq (4)), *ν*, λ_1_ and λ_2_ are fixed parameters, usually λ_1_ = λ_2_ = 1 and *ν* = 0. Fig 1 shows an example of the application of the Chan-Vese active contour curve (Eq (5)) to the problem under analysis.

### Deep CNN architecture

Deep learning is a new branch of machine learning that improves traditional shallow learning models by including multiple layers to manage and process large amounts of data. The extra layers specialize in different features during the training stage. For example, in image classification, the visual features from an input image are later combined to detect higher-order features that are relevant to the final classification. Thus, severe problems in image classification and recognition are easier to solve now [26].

The deep CNN is an exclusive deep-learning model [62, 63], the popularity of which has increased on image labeling problems. It is a multilayered approach of conventional convolutional neural networks that include an input layer, a set of hidden layers (which could vary depending on the network architecture from two to hundreds of layers), and an output layer, usually, a fully connected layer. Each hidden layer is based on the CNN architecture core, consisting of at least the convolutional and max-pooling layers. Other configurations extend the basic scheme by adding dropout and flatten layers [64]. This multilayer structure enables the network to learn different data abstractions while transitioning from layer to layer until reaching the output result [20].

### Proposed detector

**Fig 2:**
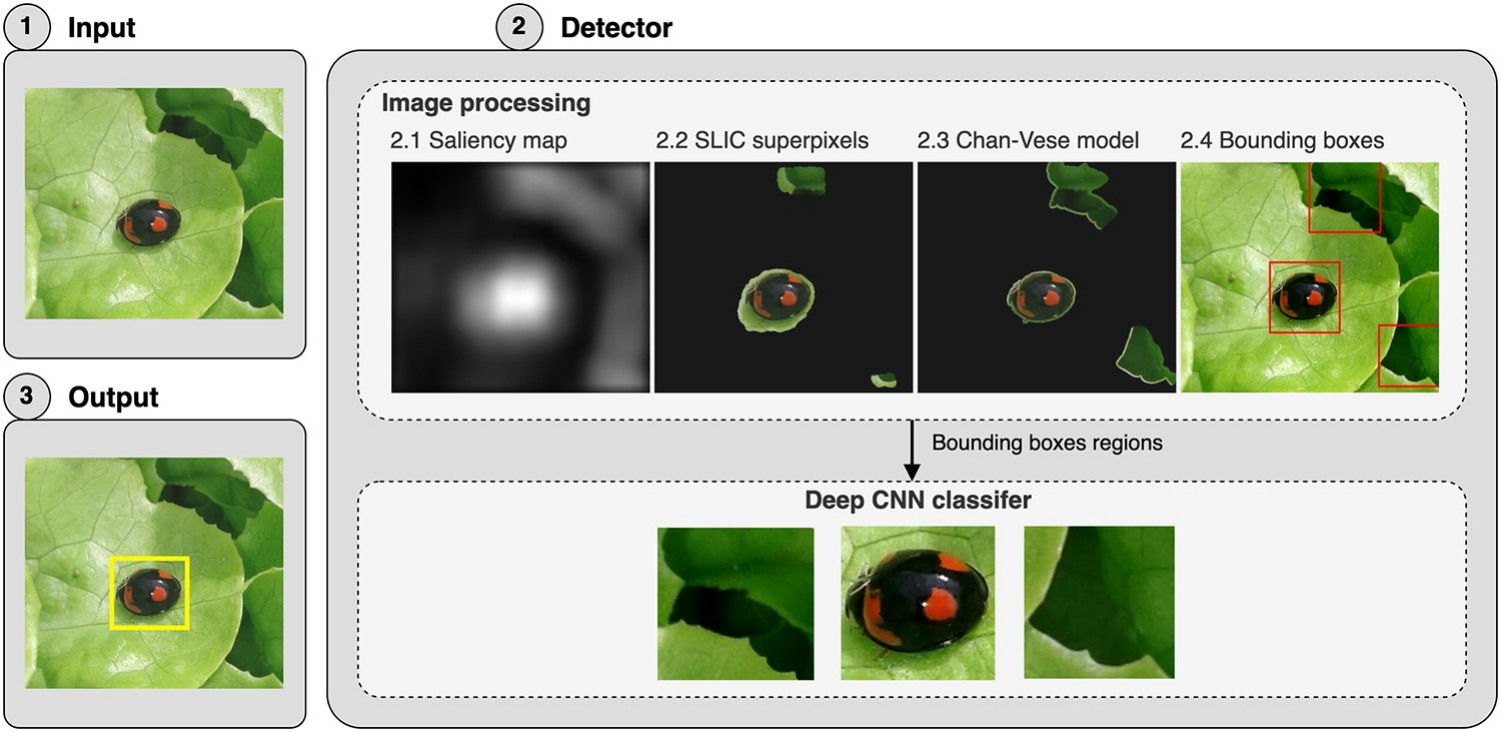
Workflow of the proposed detector.

The proposed detector is based on the combination of digital image processing and deep-learning techniques to enhance and classify the objects in the image as the desired object, i.e., the ladybird beetles (see Fig 2, step 1). In this setting, we developed two modules separated by tasks: image processing to generate bounding boxes with possible ladybird beetle inside and a deep CNN classifier to determine which of the generated bounding boxes have ladybird beetles. Once both modules are integrated, it is possible to detect the ladybird beetles in the input image (see Fig 2, step 3).

The image processing tasks include determining the image saliency map to highlight important areas with possible ladybird beetles (see Fig 2, step 2.1). The superpixels segmentation method is then applied to extract the regions detected in the image (see Fig 2, step 2.2). The Chan-Vese active contour model then obtains the final segmentation to refine the segmented areas using the superpixels method (see Fig 2, step 2.3). Bounding boxes are then generated to enclose (mostly in a rectangular shape) the segmented areas of the previous step (see Fig 2, step 2.4). On the other hand, the classifier is based on a deep CNN architecture to avoid introducing false positives in the bounding boxes classification, e.g., boxes detected but without containing ladybird beetles. A detailed description of these modules follows.

#### Image processing

This step aims to define bounding boxes that show the location of possible beetle specimens within the total image. The proposed method employed several image segmentation techniques and morphological operations to achieve this goal. First, the saliency map method is applied to the input image to obtain a gray-level scale image highlighting the most relevant areas. In some cases, these areas are big and connected between them, making it challenging to delimit regions of interest (ROI) possibly containing ladybird beetles. Thus, an initial statistical analysis of the pixel intensity values concludes that only pixels above a threshold value of 90 units in the saliency map are sufficient to produce ROIs with smaller areas. Besides, the segmentation of detected ROIs was improved by applying the Chann-Vese active contour model with 50 iterations and a dilate-based morphological operation with a disk-based structuring element with a radius of 10 units. As a result, well-delimited ROIs were obtained, which enclose possible ladybird beetles candidates (see Fig 2, step 2.1). Subsequently, the SLIC superpixels method was used to create a new and more precise mask of the ladybird beetles within each segmented ROIs. This approach can distinguish between the object foreground and background in scenes where both aspects contrast, a frequent scenario found in our database (see Fig 2, step 2.2).

Finally, we repeated the Chann-Vese active contour model application with 100 iterations and a morphological dilate operation with a disk-based structuring element with a radius of 5 units pixels to achieve the final segmentation of the ladybird beetle mask inside the ROIs (see Fig 2, step 2.3). The resulting segmented masks were used to generate the bounding boxes (mainly square shape), which are the output of this module and that are used to feed the deep CNN classifier (see Fig 2, step 2.4 red boxes).

#### Deep CNN classifier

**Fig 3:**
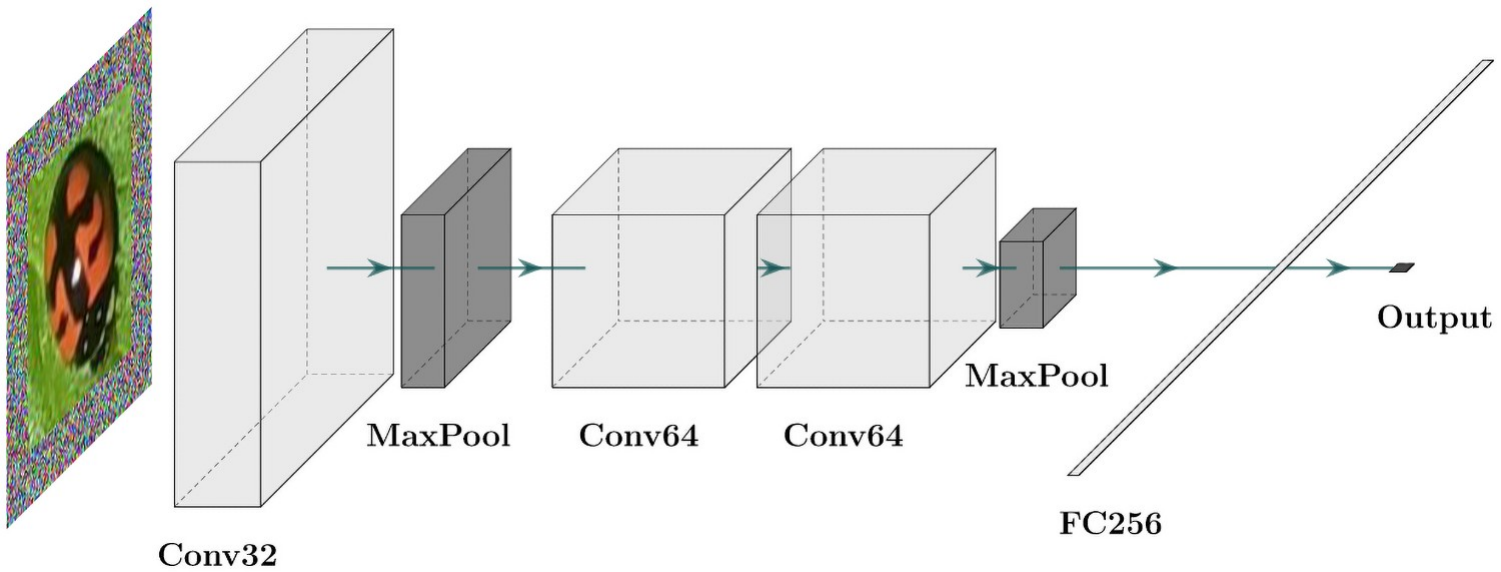
Proposed DCNN1 model.

The final stage of the proposed method uses a deep CNN classifier to determine whether or not the generated bounding boxes (output of the image processing module) contain ladybird beetles (see Fig 2 step 2). Thus, we adopted the standard deep CNN architecture to build four possible classification models, named DCNN1, DCNN2, DCNN3, and DCNN4, to fulfill the classification task. However, only the best one is integrated into the final proposed detector. These models were built based on the main architecture components variation, i.e., on the number of convolutional layers and their respective number of filters, the number of pooling layers, and their location within the network. We focus our description on the DCNN1 classification model to explain this stage better, as shown in Fig 3.

The bounding box regions generated in the previous step are now used to feed the first convolutional layer, composed of 32 convolutional filters with a [5 × 5] kernel size. This layer aims to predict the input sample class probabilities by creating a feature map representation computed by the filter structure. Subsequently, the feature map enters a max-pooling layer with a [3 × 3] size to reduce irrelevant features (information) while retaining the relevant ones. The reduced feature space is then used to feed two more convolutional layers (64 filters each with a kernel size of [3 × 3]) and a pooling layer with the same configuration as the previous one. This second convolutional module concentrates the most relevant (important) features to classify the input sample. Finally, a fully connected layer consisting of two dense layers with 256 and 1 neurons is used to determine the final output. The first layer employs the rectified linear unit (ReLU) activation function to convert and reduce the bi-dimensional input feature space into a single feature vector with corresponding weights. The output layer uses the sigmoid activation function and provides the final (binary) classification for a given feature vector.

The other implemented models (DCNN2, DCNN3, and DCNN4) follow the same deep CNN base architecture, varying the layers configurations and employing the same fully connected layer configuration as in the DCNN1 model. Additionally, all the models include an input layer with a size of [144 × 144]. Table 1 shows an overview of the core structure of proposed deep models.

**Table 1:** Core structure of the proposed deep CNN models.

DCNN1	DCNN2	DCNN3	DCNN4
Conv.(32)+Kernel (5 × 5)	Conv.(32)+Kernel (5 × 5)	Conv.(32)+Kernel (5 × 5)	Conv.(32)+Kernel (5 × 5)
max-pooling (3 × 3)	max-pooling (3 × 3)	max-pooling (3 × 3)	max-pooling (3 × 3)
Conv.(64)+Kernel (5 × 5)	Conv.(32)+Kernel (5 × 5)	Conv.(32)+Kernel (5 × 5)	Conv.(32)+Kernel (5 × 5)
Conv.(64)+Kernel (5 × 5)	max-pooling (3 × 3)	max-pooling (3 × 3)	max-pooling (3 × 3)
max-pooling (3 × 3)	Conv.(64)+Kernel (5 × 5)	Conv.(32)+Kernel (5 × 5)	Conv.(64)+Kernel (5 × 5)
fully connected (256, 1)	Conv.(64)+Kernel (5 × 5)	max-pooling (3 × 3)	Conv.(64)+Kernel (5 × 5)
	max-pooling (3 × 3)	Conv.(64)+Kernel (5 × 5)	max-pooling (3 × 3)
	fully connected (256, 1)	Conv.(64)+Kernel (5 × 5)	Conv.(64)+Kernel (5 × 5)
		max-pooling (3 × 3)	Conv.(64)+Kernel (5 × 5)
		fully connected (256, 1)	max-pooling (3 × 3)
			Fully connected (256, 1)

Conv.- convolutional layer

### Experimental setup

This section defines the experimental setup used to evaluate the proposed method of detecting ladybird beetle species in random environments. Data preparation, training and test partitions, models optimization, assessment metrics, and model selection are the essential aspects explained in the following subsections.

#### Data preparation

**Fig 4:**
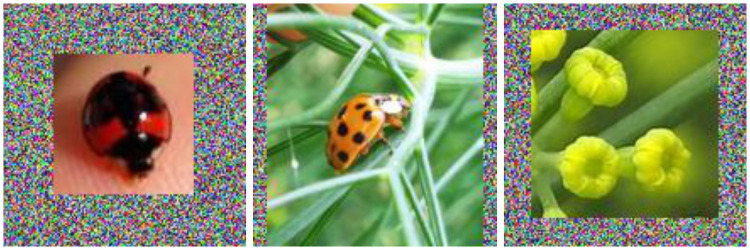
An example of a padding operation on bounding boxes regions smaller than the required input dimension.

The output of the image-processing module is a set of bounding boxes that may contain or not ladybird beetles, and each box may have different dimensions (first stage of the proposed detector). Therefore, it was mandatory to prepare them to meet the required settings of the learning process in the deep CNN classifier (second stage of the proposed detector). Bounding boxes labeling and padding tasks were done as follows:

- Manual labeling: the employed image processing algorithms are prone to introduce false-positive regions such as those containing flowers, leaves, unrelated insect specimens, amongst others. Due to their functionalities, they are bound to highlight any area with a possible object. Thus, we manually inspected and labeled the bounding boxes into two categories: boxes with and without ladybird beetle specimen, i.e., positive and negative samples.
- Padding: the proposed deep CNN models are constrained to an input canvas (image) with a size of [144 × 144] pixels. Thus, if any dimension of the bounding box is larger than 144 pixels, we isotropically resize it to fit in the required canvas dimension. Otherwise, we centered it in the canvas and started adding white noise (random values for every pixel channel) to every empty pixel in the canvas, as shown in Fig 4.

After completing both tasks, we used the labeled and padded bounding boxes to form an experimental data set for training and testing the proposed deep CNN models.

#### Training and test partitions

We apply a stratified 5-fold cross-validation method [65] on the experimental data set to form disjoint training and test partitions and guarantee the class representation on each partition. This process was repeated ten times with different random initialization seeds to reflect better the classification capabilities of the model for previously unseen data.

#### Models optimization

For all the models, we optimized the *L*2 regularization parameter in the values of 0.001, 0.003, and 0.005 to prevent overfitting during the model training. The number of training iterations (epochs) was optimized in the range from 20 to 50, with an increment of 10 units. Other hyperparameters were kept constant throughout the optimization, such as a learning rate of 3 × 10^−4^, batch size of 128, convolutional kernel size of [5 × 5] with a single stride, max-pooling kernel size of [3 × 3] with a stride of 3 units, the *same* padding type for all convolutional layers, and the *adam* optimizer, which is based on adaptive estimation of lower-order moments [66].

#### Assessment metrics

Regarding the bounding boxes generation, we computed the ACC metric to evaluate the image processing module (first stage of the proposed detector). Since it is possible to have several bounding boxes per image, we considered a true positive generation if the ladybird beetle specimen is located inside a bounding box. Otherwise, we considered it as a false positive. The error rate metric associated with the ACC performance was also calculated to support the discussion of the proposed detector limitations. In this case, the error rate metric measures the percentage of bounding boxes generated with incomplete ladybird beetles inside.

Concerning the deep CNN-based classification (second stage of the proposed detector), we calculated the mean of the AUC (area under the receiver operating characteristic curve), ACC, precision (PRE), and recall (REC) to assess the effectiveness of all classification models on the experimental data set over 50 runs. Also, we performed a statistical comparison between classification models using the Wilcoxon statistical test [36] with *α* = 0.05 to determine if there is any statistically significant difference among models. Despite computing several validation metrics, we supported the discussion of results using the mean of the AUC metric.

#### Model selection

Since the proposed detector explored several classification models, a golden rule was set for selecting the best classification scheme. Thus, we follow first the model that provided the higher mean of AUC performance statistically at *p* < 0.05, and second, if there was a tied performance in the resulting scores, the model with the most straightforward architecture was selected and implemented within the proposed detector.

All deep CNN classifiers were implemented, trained, and evaluated in Python language version 3.6.9 [67] using Keras [68] (MXNET backend [69]) and the *scikit-learn (SKlearn)* library [70].

## Results and discussion

The performance of the proposed detector is next analyzed by considering its two internal stages results separately. First, the results from the image processing module (first stage) are presented. Then, the deep CNN model classification performance is evaluated on an experimental data set containing the bounding boxes regions produced by the first stage of the proposed detector.

### Performance of bounding box generation

**Fig 5:**
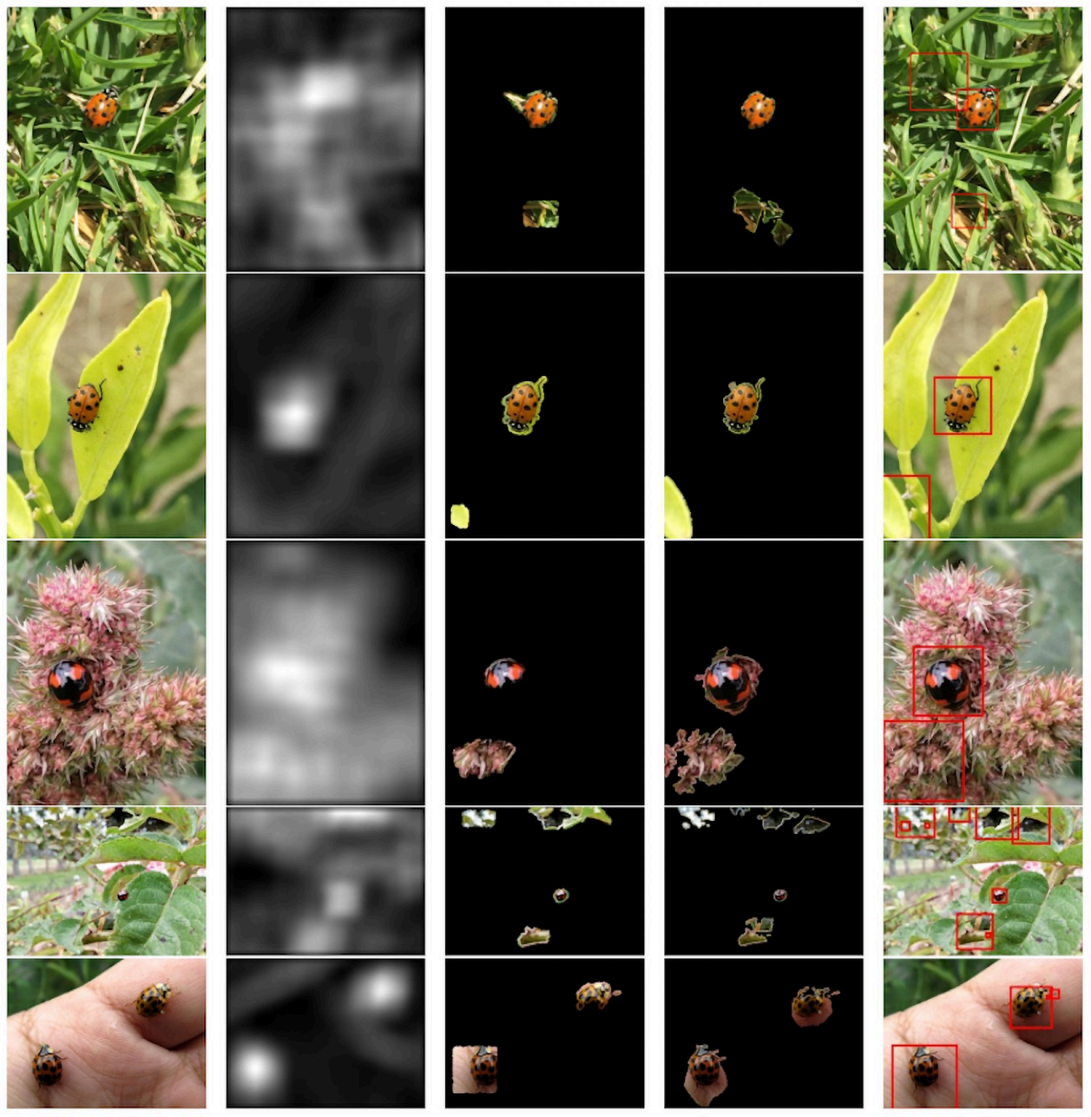
Successful performance examples of the image processing module (first stage of the proposed detector) in different environments. From left to right, original image, saliency map, SLIC superpixels segmentation, Chan-Vese active contour, and final bounding boxes generated.

A total of 2, 300 images with ladybird beetle species from the *iNaturalist* project were used to feed the proposed detector. It was possible to generate a bounding box around the ladybird beetles in the images in most cases, demonstrating successful performance. The obtained ACC score of 92% corroborated the satisfactory results. Some examples of successful detection are shown in Fig 5.

From this figure, it is possible to observe that the saliency map step (second column) correctly revealed the ladybird beetle area in the image. However, it also spotlighted other non-desired regions, which could produce bounding boxes without ladybird beetles. This effect is present because this method is intended to highlight the most relevant image regions according to the pixel quality, which is greater where the focus of attention is given in the scene. Similarly, the SLIC superpixel segmentation method (third column) correctly segmented the saliency map discovered areas. Even when some of them do not contain ladybird beetles, after that, the Chan-Vese model (fourth column) refined the ladybird beetle contour inside the segmented areas. It is also possible to notice that this method has low performance when the target object is missing. This behavior was expected since the Chan-Vese model started the segmentation process (curve deformation) on areas without any ladybird beetles. Finally, the bounding boxes were correctly generated (fifth column), enclosing the ladybird beetles and other non-desired regions, which were wrongly revealed by the saliency map method.

It should be noted that this part of the proposed detector does not consider any knowledge about the ladybird beetles. Therefore, as long as the saliency map method highlights correct areas, the remaining processes will perform better and vice versa. For instance, the ladybird beetles were correctly enclosed in common backgrounds such as grass or leaves (see Fig 5, first and second rows). Also, it worked well in more homogeneous contexts such as flowers and hands red, where the background colors are very similar to some parts of the ladybird beetle specimen (see Fig 5, third and fifth rows). Even in scenarios where the ladybird beetles are tiny and frequent (more than one) objects in the image, they were precisely enclosed by the proposed detector (see Fig 5, fourth and fifth rows).

### Performance of deep CNN models

The image processing module of the proposed detector (first stage) provided a total of 9,925 bounding box regions, which were processed to form an experimental data set distributed in 3, 024 and 6, 901 bounding boxes with and without ladybird beetles, respectively. This dataset was used together with the 5-fold cross-validation method to feed the proposed deep CNN models. The obtained classification performance highlighted quality results, as shown in Table 2.

**Table 2:** Performance results of proposed deep CNN models.

Arquitecture	Reg.	Epochs	AUC±SD	ACC±SD	PRE±SD	REC±SD	Wil. (*α* = 0.05)
DCNN1	0.001	20	0.968 ± 0.005	91.88 ± 0.008	0.901 ± 0.031	0.827 ± 0.030	*p* < 0.05
	0.003	20	0.963 ± 0.005	91.09 ± 0.009	0.891 ± 0.041	0.809 ± 0.049	*p* < 0.05
	0.005	20	0.960 ± 0.006	90.63 ± 0.010	0.882 ± 0.037	0.802 ± 0.039	*p* < 0.05
	0.001	30	0.971 ± 0.004	92.34 ± 0.007	0.907 ± 0.031	0.836 ± 0.037	*p* < 0.05
	0.003	30	0.968 ± 0.004	91.74 ± 0.009	0.906 ± 0.040	0.817 ± 0.044	*p* < 0.05
	0.005	30	0.964 ± 0.005	91.21 ± 0.009	0.905 ± 0.038	0.801 ± 0.046	*p* < 0.05
	0.001	40	0.972 ± 0.004	92.66 ± 0.007	0.909 ± 0.030	0.845 ± 0.036	*p* < 0.05
	0.003	40	0.970 ± 0.005	92.26 ± 0.007	0.919 ± 0.028	0.818 ± 0.038	*p* < 0.05
	0.005	40	0.966 ± 0.004	91.56 ± 0.009	0.903 ± 0.039	0.811 ± 0.051	*p* < 0.05
	0.001	50	0.972 ± 0.005	92.79 ± 0.006	0.905 ± 0.031	0.849 ± 0.038	*p* < 0.05
	0.003	50	0.971 ± 0.004	92.31 ± 0.008	0.920 ± 0.026	0.817 ± 0.049	*p* < 0.05
	0.005	50	0.968 ± 0.004	91.85 ± 0.006	0.905 ± 0.034	0.821 ± 0.035	*p* < 0.05
DCNN2	0.001	20	0.972 ± 0.004	92.42 ± 0.007	0.920 ± 0.031	0.825 ± 0.043	*p* < 0.05
	0.003	20	0.968 ± 0.004	91.78 ± 0.009	0.909 ± 0.038	0.817 ± 0.052	*p* < 0.05
	0.005	20	0.965 ± 0.005	91.16 ± 0.011	0.895 ± 0.050	0.813 ± 0.060	*p* < 0.05
	0.001	30	0.975 ± 0.004	93.01 ± 0.007	0.932 ± 0.024	0.831 ± 0.039	*p* < 0.05
	0.003	30	0.971 ± 0.004	91.97 ± 0.008	0.923 ± 0.033	0.809 ± 0.049	*p* < 0.05
	0.005	30	0.967 ± 0.005	91.58 ± 0.014	0.914 ± 0.044	0.803 ± 0.062	*p* < 0.05
	**0.001**	**40**	**0.977** **±** **0.003**	**93.37** **±** **0.007**	**0.932** **±** **0.025**	**0.843** **±** **0.036**	**p = 0.57**
	0.003	40	0.973 ± 0.005	92.59 ± 0.009	0.925 ± 0.029	0.828 ± 0.045	*p* < 0.05
	0.005	40	0.970 ± 0.004	91.76 ± 0.010	0.911 ± 0.040	0.816 ± 0.055	*p* < 0.05
	0.001	50	0.978 ± 0.004	93.53 ± 0.008	0.939 ± 0.025	0.845 ± 0.038	−
	0.003	50	0.974 ± 0.005	92.73 ± 0.009	0.933 ± 0.029	0.821 ± 0.043	*p* < 0.05
	0.005	50	0.970 ± 0.005	92.00 ± 0.009	0.920 ± 0.038	0.811 ± 0.053	*p* < 0.05
DCNN3	0.001	20	0.968 ± 0.005	92.02 ± 0.008	0.897 ± 0.033	0.836 ± 0.031	*p* < 0.05
	0.003	20	0.960 ± 0.008	90.79 ± 0.013	0.878 ± 0.046	0.826 ± 0.039	*p* < 0.05
	0.005	20	0.954 ± 0.007	89.81 ± 0.020	0.847 ± 0.057	0.825 ± 0.049	*p* < 0.05
	0.001	30	0.973 ± 0.005	92.73 ± 0.008	0.920 ± 0.028	0.833 ± 0.040	*p* < 0.05
	0.003	30	0.968 ± 0.005	91.78 ± 0.009	0.902 ± 0.050	0.818 ± 0.049	*p* < 0.05
	0.005	30	0.960 ± 0.009	89.04 ± 0.081	0.854 ± 0.089	0.835 ± 0.056	*p* < 0.05
	0.001	40	0.976 ± 0.004	93.25 ± 0.006	0.928 ± 0.024	0.846 ± 0.030	*p* = 0.08
	0.003	40	0.970 ± 0.005	92.07 ± 0.010	0.911 ± 0.037	0.823 ± 0.046	*p* < 0.05
	0.005	40	0.964 ± 0.007	90.94 ± 0.023	0.887 ± 0.053	0.821 ± 0.053	*p* < 0.05
	0.001	50	0.977 ± 0.005	93.42 ± 0.010	0.930 ± 0.031	0.847 ± 0.034	*p* = 0.96
	0.003	50	0.971 ± 0.006	91.91 ± 0.021	0.916 ± 0.058	0.820 ± 0.047	*p* < 0.05
	0.005	50	0.965 ± 0.008	89.21 ± 0.092	0.882 ± 0.105	0.822 ± 0.060	*p* < 0.05
DCNN4	0.001	20	0.966 ± 0.005	91.54 ± 0.008	0.892 ± 0.041	0.826 ± 0.044	*p* < 0.05
	0.003	20	0.954 ± 0.007	90.32 ± 0.012	0.853 ± 0.047	0.825 ± 0.056	*p* < 0.05
	0.005	20	0.951 ± 0.007	89.89 ± 0.010	0.838 ± 0.043	0.829 ± 0.043	*p* < 0.05
	0.001	30	0.971 ± 0.005	92.26 ± 0.007	0.913 ± 0.033	0.825 ± 0.042	*p* < 0.05
	0.003	30	0.962 ± 0.007	90.89 ± 0.015	0.889 ± 0.043	0.814 ± 0.048	*p* < 0.05
	0.005	30	0.957 ± 0.006	90.58 ± 0.008	0.863 ± 0.042	0.825 ± 0.047	*p* < 0.05
	0.001	40	0.973 ± 0.004	92.67 ± 0.007	0.923 ± 0.027	0.830 ± 0.052	*p* < 0.05
	0.003	40	0.966 ± 0.006	91.63 ± 0.008	0.902 ± 0.033	0.819 ± 0.048	*p* < 0.05
	0.005	40	0.959 ± 0.012	90.61 ± 0.031	0.863 ± 0.130	0.803 ± 0.125	*p* < 0.05
	0.001	50	0.975 ± 0.004	93.08 ± 0.009	0.930 ± 0.026	0.835 ± 0.037	*p* < 0.05
	0.003	50	0.967 ± 0.007	91.50 ± 0.018	0.917 ± 0.030	0.811 ± 0.054	*p* < 0.05
	0.005	50	0.963 ± 0.005	91.31 ± 0.009	0.875 ± 0.131	0.803 ± 0.123	*p* < 0.05

Reg.—*L*2 regularization parameter; AUC, ACC, PRE and REC—mean of: AUC, ACC, PRE, and REC metrics over 50 runs; SD—standard deviation; underlined AUC value is the Wilcoxon test pivot value; selected model in bold.

From this table, it is possible to read that all models obtained AUC mean scores over the 0.95, which means that the proposed four deep architectures and employed configurations provided successful classification models. The higher mean of AUC score of 0.978±0.03 was reached by the DCNN2 model with a *L*2 regularization value of 0.001 and 50 epochs. However, this performance was not statistically superior (*α* = 0.05) to the classification model based on the DCNN2 model with a *L*2 regularization value of 0.001 and 40 epochs (AUC = 0.977±0.003, *p* = 0.574), and the two others models using the DCNN3 with the same *L*2 regularization value of 0.001, but with 40 (AUC = 0.976±0.004, *p* = 0.076) and 50 (AUC = 0.977±0.005, *p* = 0.959) epochs, respectively.

It should be noted that none of the DCNN1 and DCNN4 models were good candidates when compared to those from the DCNN2 and DCNN3 models. This behavior is related to the core structure of these architectures. The DCNN1 model used three convolutional layers (see Table 1) that seem insufficient to learn the needed abstractions from the images of the random environment, including the ladybird beetles details. On the other hand, the DCNN4 model employed six convolutional layers (see Table 1) that could resemble an efficient architecture, but it was not. In this case, the use of two blocks of convolutional layers with 64 filters each demands more samples (bounding boxes with ladybird beetles) to learn and extract adequate image features. That explains the reason why some models from this architecture were the only reaching AUC mean scores around the 0.95 (worst scores). In contrast, the DCNN2 and DCNN3 models were in the middle of explored architectures with acceptable combinations of convolutional layers that enable them as good classification models to tackle the problem under analysis.

**Fig 6:**
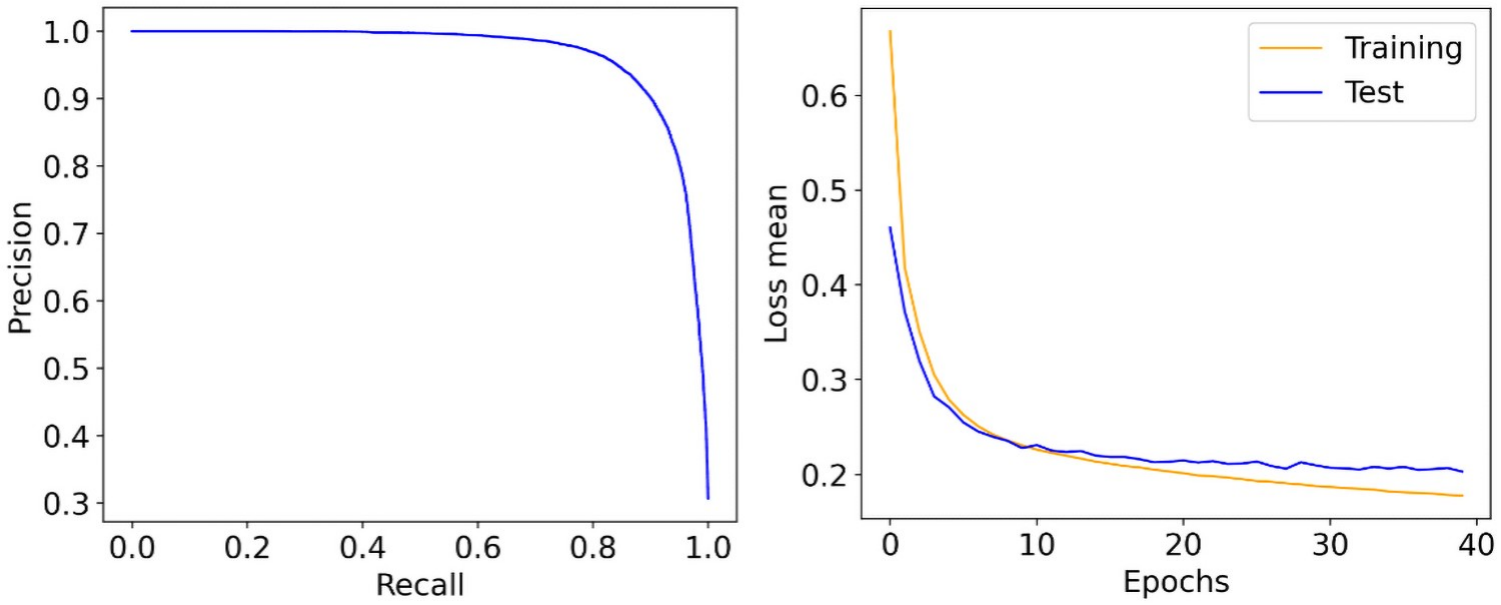
Performance of the selected deep CNN model based on the P-R curve (left) and cross-entropy loss function during the training and test stages (right).

According to the model selection criteria, the DCNN2 model with a *L*2 regularization value of 0.001 and 40 epochs was considered the best model to integrate into the second stage of the proposed detector. It did not obtain the highest mean AUC score, but it was statistically similar in terms of performance with a mean of AUC = 0.977±0.003 (*p* = 0.574). It was the most straightforward model among those with similar performance. It did not incur overfitting during the training process while maintaining good precision and recall performance during the test (see Fig 6, left panel). Moreover, we can observe that the cross-entropy red-based loss function describes a similar performance for both the training and test curves. This behavior guarantees the good generalization power of the model when classifying new unseen data (see Fig 6, right panel).

### Limitations of the proposed detector

**Fig 7:**
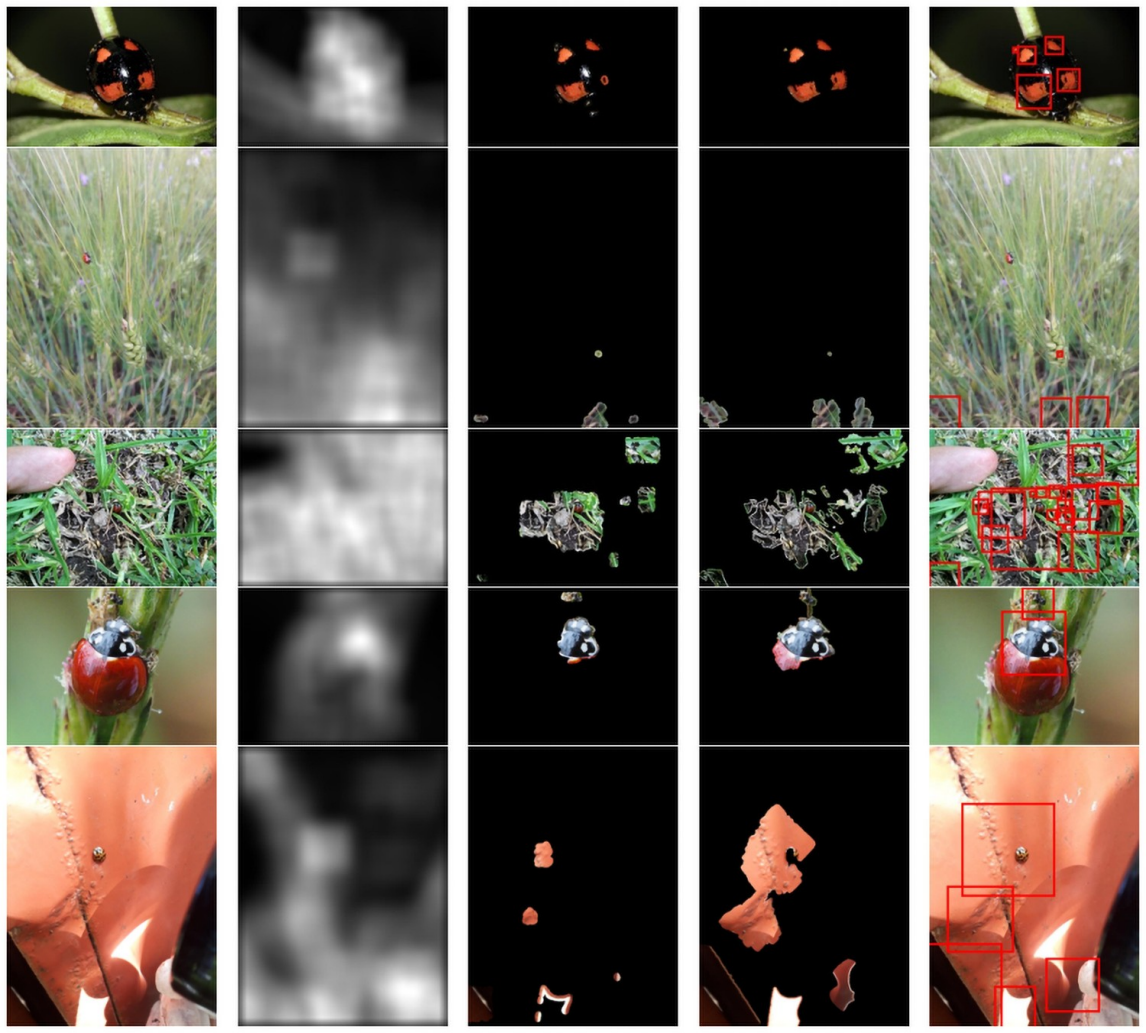
Unsuccessful performance examples of the image processing module (first stage of the proposed detector) in different environments. From left to right, original image, saliency map, SLIC superpixels segmentation, Chan-Vese active contour, and final bounding boxes generated.

The principal limitations of the present proposal are associated with the image processing module of the proposed detector. Even though the bounding boxes generation part performed well in almost all cases, there was an error rate of 8% related to some complex scenarios where the saliency map failed to correctly estimate the ladybird beetle area, as shown in Fig 7. For example, this could happen when the ladybird beetle contour is too similar to the surrounding background. In that case, the detector tends to find only the ladybird beetle patterns that contrast with the background, e.g., the orange spots (see Fig 7, first row). In such a case, the ladybird beetle sample black patterns are highly correlated to the background intensity, thus provoking a missing detection and wrong bounding boxes generation.

Other challenging scenarios are related to the size and out of focus of the ladybird beetles in the scene. Both cases come from randomly taken images in uncontrolled environments (poor lightning conditions, objects too far from the lens, hand-taken picture with low camera shutter speed, among others). Under these situations, the saliency map highlighted several random regions in the image, which sometimes could contain the ladybird beetles or not (see Fig 7, second, third, and fifth rows). In opposite, when the ladybird beetle size occupies a significant portion of the image (macro photo), usually the generated bounding box is incomplete, i.e., less than a half of the correct size (see Fig 7, fourth row). In this case, despite the excellent performance of the saliency map method, the ladybird beetle was not correctly enclosed by the bounding box. This lousy result is linked to the insufficient number of iterations employed by the Chan-Vese active contour. We used 50 and 100 iterations for the active-contour model first and second applications, respectively. These values were enough for most small-medium samples but insufficient for bigger ones. Except for the last case, the main drawbacks of this part of the proposed detector are correlated with the saliency-map low performance. As we mentioned before, this method is the base of the image processing module, and any failure in its application will interfere with the remaining processes.

Regarding the deep CNN classifier, the main limitation is linked to the data (bounding boxes) preparation before training the classifier. As we mentioned before, the bounding boxes contain variable sizes that need to be standardized to a fixed input size in the classification model. This classifier uses supervised learning and should be retrained at some point in time to improve the classification performance. Thus, the bounding box labeling and padding tasks become mandatory during the training process.

## Conclusion

We proposed a two-stage approach for the automatic detection of ladybird beetles in random environment images. First, an image processing module composed of the saliency map, SLIC superpixels segmentation, and active contour methods allowed us to generate bounding boxes with possible ladybird beetles. Subsequently, a deep CNN-based classifier determines only the bounding boxes with ladybird beetles as the final output. The proposed method was validated on a data set of 2, 300 images from Ecuador and Colombia regions in the *iNaturalist* project highlighting an ACC score of 92% and an AUC score of 0.977 for the bounding box generation and classification tasks, respectively. These successful results enable the proposed detector as a valuable tool for helping specialists in the ladybird beetle detection problem.

As future work, we plan to improve the image processing module by combining the saliency map method with an adaptive local pattern analysis (ladybird neighborhood inspection) towards the correct bounding box generation. In this sense, we will overcome most of the limitations of this work by experimenting with more extensive ladybird beetle image databases to validate detection performance deeply. We also want to explore the use of deep learning models in the whole workflow to benchmark the detection performance and determine the best solution to be implemented in a future mobile device application.
